# The Photosynthetic Efficiency and Carbohydrates Responses of Six Edamame (*Glycine max*. L. Merrill) Cultivars under Drought Stress

**DOI:** 10.3390/plants11030394

**Published:** 2022-01-31

**Authors:** Jeremiah M. Hlahla, Mpho S. Mafa, Rouxléne van der Merwe, Orbett Alexander, Mart-Mari Duvenhage, Gabre Kemp, Makoena J. Moloi

**Affiliations:** 1 Department of Plant Sciences-Botany Division, Faculty of Natural and Agricultural Sciences, University of the Free State, 205 Nelson Mandela Drive, Park West, Bloemfontein 9301, South Africa; hlahlajer@gmail.com (J.M.H.); vandermerwer@ufs.ac.za (R.v.d.M.); 2 Carbohydrates and Enzymology Laboratory (CHEM-LAB), Department of Plant Sciences-Botany Division, University of the Free State, 205 Nelson Mandela Drive, Park West, Bloemfontein 9301, South Africa; mafams@ufs.ac.za; 3 Department of Chemistry, University of the Free State, 205 Nelson Mandela Drive, Park West, Bloemfontein 9301, South Africa; alexandero@ufs.ac.za; 4 Department of Physics, University of the Free State, 205 Nelson Mandela Drive, Park West, Bloemfontein 9301, South Africa; devenhagem@ufs.ac.za; 5 Department of Microbiology and Biochemistry, University of the Free State, 205 Nelson Mandela Drive, Park West, Bloemfontein 9301, South Africa; kempg@ufs.ac.za

**Keywords:** carbohydrates, carotenoids, drought, edamame, photosynthesis, stomatal conductance, hemicellulose, lignin

## Abstract

Vegetable-type soybean, also known as edamame, was recently introduced to South Africa. However, there is lack of information on its responses to drought. The aim of this study was to investigate the photosynthetic efficiency and carbohydrates responses of six edamame cultivars under drought stress. Photosynthetic efficiency parameters, including chlorophyll fluorescence and stomatal conductance, were determined using non-invasive methods, while pigments were quantified spectrophotometrically. Non-structural carbohydrates were quantified using Megazyme kits. Structural carbohydrates were determined using Fourier-transform infrared spectroscopy (FTIR) and X-ray diffraction (XRD). Drought stress significantly increased the Fv/Fm and PIabs of AGS429 and UVE17 at pod filling stage. Chlorophyll-a, which was most sensitive to drought, was significantly reduced in AGS429 and UVE17, but chlorophyll-b was relatively stable in all cultivars, except UVE17, which showed a significant decline at flowering stage. AGS354 and AGS429 also showed reduced chlorophyll-b at pod filling. UVE17 showed a significant reduction in carotenoid content and a substantial reduction in stomatal conductance during pod filling. Drought stress during pod filling resulted in a significant increase in the contents of trehalose, sucrose and starch, but glucose was decreased. Chlorophyll-a positively correlated with starch. The FTIR and XRD results suggest that the cell wall of UVE14, followed by UVE8 and AGS429, was the most intact during drought stress. It was concluded that carotenoids, stomatal conductance, starch and hemicellulose could be used as physiological/biochemical indicators of drought tolerance in edamame. This information expands our knowledge of the drought defense responses in edamame, and it is essential for the physiological and biochemical screening of drought tolerance.

## 1. Introduction

Drought stress, which is accelerated by anthropogenic climate change, is a multidimensional environmental stress that affects growth and development of plants by altering their normal metabolic activities, resulting in poor crop yield and quality [1]. Vegetable-type soybean (*Glycine max* L. Merrill), also called edamame or large-seeded soybean, is a water-demanding crop. Its yield can be reduced with up to 40% when subjected to drought stress [2]. Edamame originated in South-East Asia, but its cultivation expanded to many countries across the world due to its high nutritional value. This crop is known for its high contents of protein, edible oil, isoflavanones and dietary fiber [2,3]. Since edamame has a short life cycle and can be grown at least four times a year under favorable conditions, it could generate income for small- and large-scale farmers [4]. Drought has the greatest negative impact in Sub-Saharan Africa, resulting in huge loss in crop and total crop revenues, which negatively affect small-scale farmers [5]. The effect of drought directly contributes to nutritional insecurity in southern Africa, hence the introduction of crops such as edamame that have a high nutritional value [4]. South Africa produces about one million ton of commodity type soybean every year, but there is no recognized commercial production of vegetable-type soybean [3,6].

Generally, a plant’s tolerance responses to abiotic stress, such as drought, include modification of the morphological structure, biochemical processes and physiological features [3,7]. Moloi and Van der Merwe [3] demonstrated that different edamame genotypes displayed different biochemical responses when subjected to drought stress during the reproductive growth stages (flowering and pod filling). Drought stress may lead to reduced stomatal aperture [8], which could result in decreased rate of photosynthesis [9]. Similarly, reduced stomatal conductance (gs) of drought-stressed kidney beans (*Phaseolus vulgaris*) was followed by decreased photosynthesis rate [10]. Contrary, drought did not affect the gs in *Saussurea involucrata* transgenic cultivars [11]. Drought stress may also cause an overproduction of reactive oxygen species (ROS), which destabilize protein complexes and damage thylakoid membranes in chloroplasts [3,12]. The end result is reduced leaf surface area [8] and reduced plant growth [12,13]. Chlorophyll pigments were significantly reduced by drought stress in barley (*Hodeum vulgare*) under severe drought stress. In groundnut (*Arachis hypogaea*), chlorophyll-a (Chl-a) was more sensitive to drought stress than chlorophyll-b (Chl-b) [9,14]. An increase in the accumulation of carotenoids (CRDs) of drought-stressed transgenic tobacco (*Nicotiana tabacum*), alfalfa (*Medicago sativa*) and sweet potato (*Ipomoea batatas*) plants improved membrane stability and enhanced antioxidant activity [15,16,17]. Reduction of the photosynthetic efficiency in drought-stressed apple (*Malus pumila*) trees was associated with low Fv/Fm (which represents quantum efficiency of photosystem II), performance index absorbance (PIabs) and total performance index (PItotal) [18]. In barley cultivars, Fv/Fm only decreased under severe drought stress, but PIabs was not affected [19].

Soluble sugars, such as glucose, trehalose and sucrose, and starch accumulate in drought-stressed plants [20]. Soluble sugars improve drought tolerance in plants by preventing oxidative stress, stabilizing plasma membranes and biomolecules [3]. It was reported that in maize (*Zea mays*), the total soluble sugars content increased with a decrease in starch under drought stress. In rice (*Oryza sativa*), drought-tolerant cultivars had a higher soluble sugars content [20]. In edamame, Moloi and Van der Merwe [3] reported that the accumulation of soluble sugars is an important drought-tolerance response. Makonya et al. [7] argued that there is a direct correlation between the non-structured sugars (such as sucrose, fructose and starch) accumulation and environmental stress tolerance (drought/heat). Makonya and co-workers [7] reported that heat-resistant chickpea (*Cicer arietinum*) genotypes accumulated sucrose, fructose and starch during heat treatment in field trials, which resulted in a better yield compared to sensitive genotypes.

Additionally, modifications of structural sugars that form part of the cell wall components play an important role in strengthening plants under drought stress [21,22]. Generally, major constituents of the plant’s cell wall are cellulose [β-(1,4)-glucan], hemicelluloses (mannan, xyloglucan, xylan and mixed-linked glucan), lignin (a polymer made of phenolic compounds), trace amounts of protein and ash [23,24,25,26]. In cotton (*Gossypium hirsutum*) plants, drought stress reduced cellulose synthesis and deposition [27]. Reduced cellulose synthesis leads to thinner cell walls [28,29]. The levels of hemicelluloses may increase or decrease depending on the plant genotype and tissue [30]. The synthesis of lignin is important as it helps to strengthen the water conducting tissues and make them more hydrophobic [22], which is important in defense under drought stress [31]. Monomers of phenols and lignin are cross-linked to cell wall polysaccharides. This cross-linking makes the cell wall structure stiff [32]. In transgenic tobacco plants, lignin was found to be linked to drought resistance [32]. Drought stress significantly decreased the phenolic content in *Salix viminalis*, *Salix dasyclados*, *Vitis vinifera* and *Salix myrsinifolia*. In contrast, other studies showed that drought stress had no effect on total phenols [33].

Since soluble sugars are indicators of drought tolerance in edamame [3], it is important to specify which of these soluble sugars are involved in edamame drought tolerance. Therefore, the aim of this study was to elucidate the biochemical responses of drought tolerance in six edamame cultivars by specifically focusing on the photosynthetic efficiency, non-structural (soluble) sugars and modifications of cell wall components (including structural sugars, lignin and total phenols). These responses have not been previously reported in edamame. Responses that are only upregulated in the least sensitive genotypes could be used as biochemical markers for drought tolerance screening in the edamame breeding program.

## 2. Results

### 2.1. Photosynthetic Efficiency Indicators

**Figure 1:**
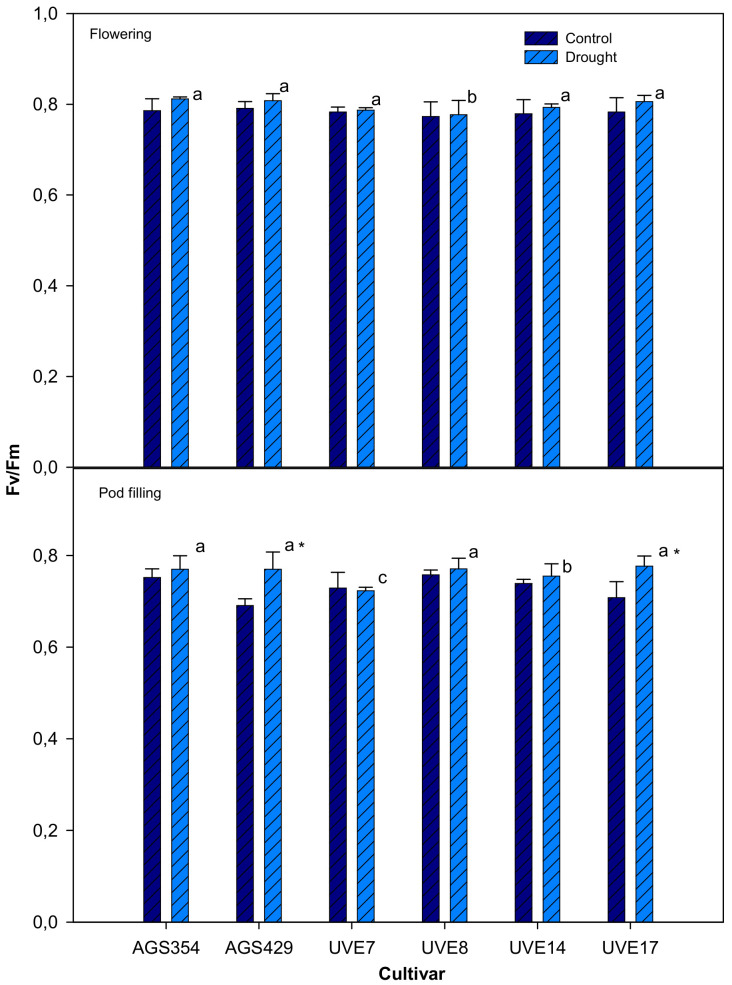
The quantum efficiency of PS II (Fv/Fm) of six edamame cultivars at flowering and pod filling stages under drought stress. Letters (a, b, and c) represent differences or similarities in Fv/Fm between cultivars. Asterisk (*) represents a significant difference under drought stress. Values represent means ± SD (*n* = 6).

Drought stress during the flowering stage did not significantly affect the PSII quantum efficiency (Fv/Fm) for all cultivars. In addition, cultivars were not significantly different under drought stress except for UVE8. Drought stress at pod filling significantly increased the Fv/Fm of AGS429 (by 10%) and UVE17 (by 9%). These two cultivars were not significantly different from each other under drought stress. For chlorophyll fluorescence, UVE14 and UVE7 were significantly different from each other from the other cultivars under drought stress. The Fv/Fm was generally higher at flowering than at pod filling (Figure 1).

**Figure 2:**
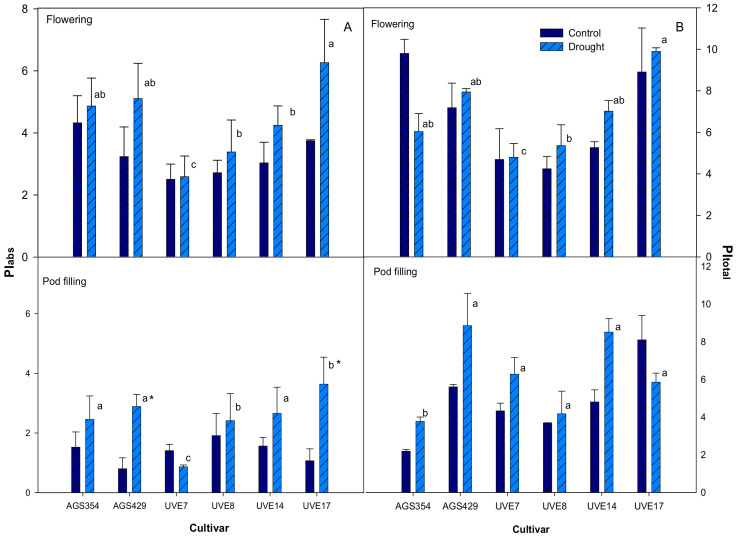
The performance indexes (PIabs) (**A**) of PSl and PSll and total Performance Index (PItotal) (**B**) of six edamame cultivars at flowering and pod filling stages under drought stress. Letters (a, b, and c) represent differences or similarities in PItotal between cultivars. Asterisk (*) represents a significant difference under drought stress. Values represent means ± SD (*n* = 6).

At flowering, PIabs increased with drought stress but the increase was not significant for all cultivars. UVE7 remained unchanged compared to the control and had the lowest PIabs. This cultivar was significantly different from the other cultivars (Figure 2A) under drought stress. At pod filling, PIabs increased with drought stress, except for UVE7, which showed a non-significant decrease with the lowest value. Drought stress significantly increased the PIabs of AGS429 by 72% and UVE17 by 71%, relative to control. UVE7 and UVE8 were significantly different from each other and to the rest of the cultivars under drought stress. UVE17 had the highest PIabs and UVE7 the lowest. PIabs was higher at flowering than at pod filling (Figure 2A). Drought stress increased PItotal for most cultivars at flowering, except for AGS354, which had a decreased PItotal compared to the control. However, the increase of PItotal in other cultivars was not significant (Figure 2B). UVE7 remained the same as the control, had the lowest PItotal and was different from other cultivars under drought stress. At pod filling, drought stress resulted in an increased PItotal for most cultivars, while the PItotal of UVE17 was inhibited. AGS354 was significantly different from other cultivars. PItotal was higher at flowering than at pod filling.

**Figure 3:**
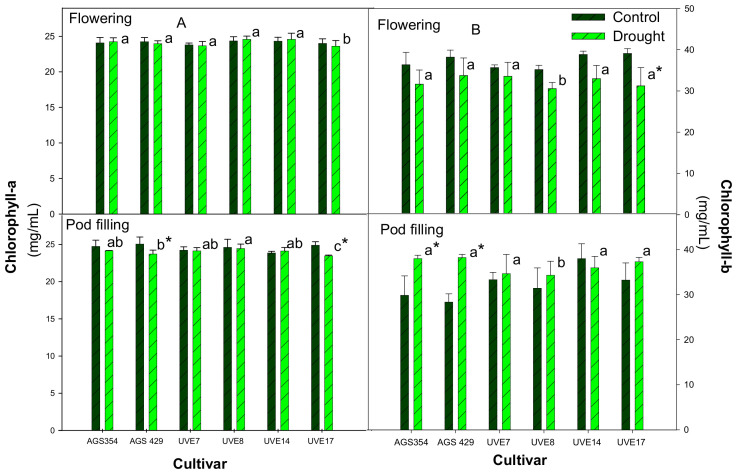
Chlorophyll-a (**A**) and Chlorophyll-b (**B**) content of six edamame cultivars at flowering and pod filling stages under drought stress. Letters (a, b, and c) represent differences or similarities in chlorophyll between cultivars. Asterisk (*) represents a significant difference under drought stress relative to controls. Values represent means ± SD (*n* = 3).

Drought stress during flowering did not have a significant effect on the Chl-a content of the six edamame cultivars. However, for UVE17, Chl-a showed a non-significant decrease under drought stress (Figure 3). In addition, this cultivar was significantly different from the other cultivars under drought stress (Figure 3A). At pod filling, drought stress resulted in a significant reduction of Chl-a for AGS429 (5%) and UVE17 (6%). No notable differences in Chl-a content were observed across flowering and pod filling. Drought stress did not have any significant effect on the Chl-b content of most drought-stressed cultivars at flowering, except UVE17, which was significantly reduced by up to 20% (Figure 3B). Drought-stressed UVE8 had the lowest Chl-b content and was significantly different from the other cultivars. At pod filling, drought stress significantly increased the Chl-b contents of AGS354 and AGS429 by 21% and 26% compared to controls, respectively. Only UVE8 was significantly different from other cultivars (shown by different letter). In general, drought-stressed cultivars had a higher Chl-b content at pod filling compared to the flowering stage.

**Figure 4:**
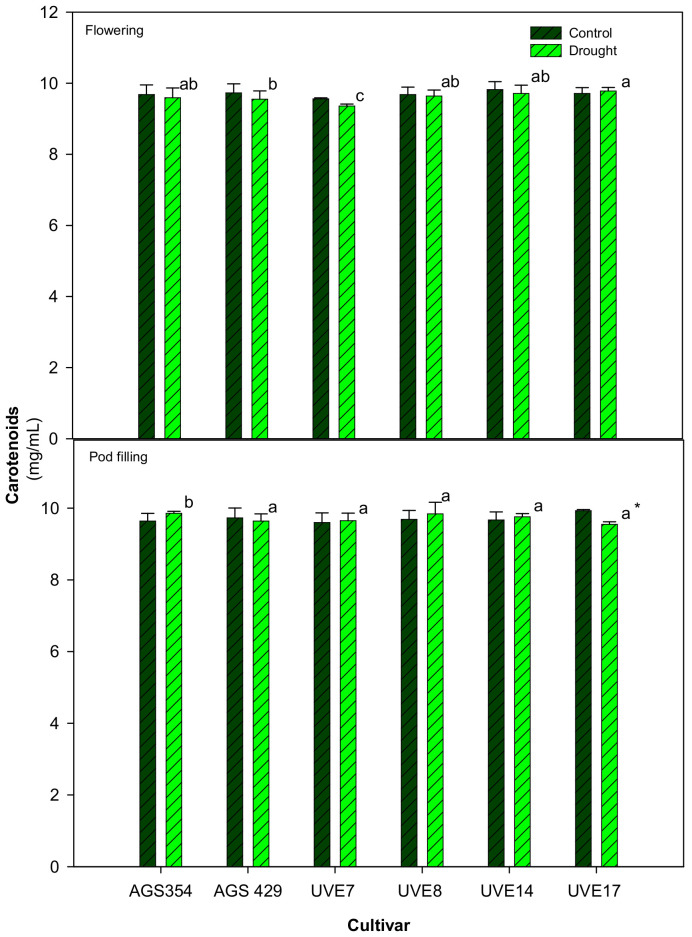
Carotenoids content of six edamame cultivars at flowering and pod filling stages under drought stress. Letters (a, b, and c) represent differences or similarities in carotenoids between cultivars. Asterisk (*) represents significant difference under drought stress. Values represent means ± SD (*n* = 3).

During the flowering stage, drought stress did not significantly affect the CRDs content across all cultivars. However, AGS429, UVE7 and UVE17 were different from each other and from other cultivars. Drought-stressed cultivars were not significantly different at pod filling. However, drought stress reduced the CRDs content of UVE17 by 4%. There were no notable differences in levels of CRDs observed across flowering and pod filling (Figure 4).

**Figure 5:**
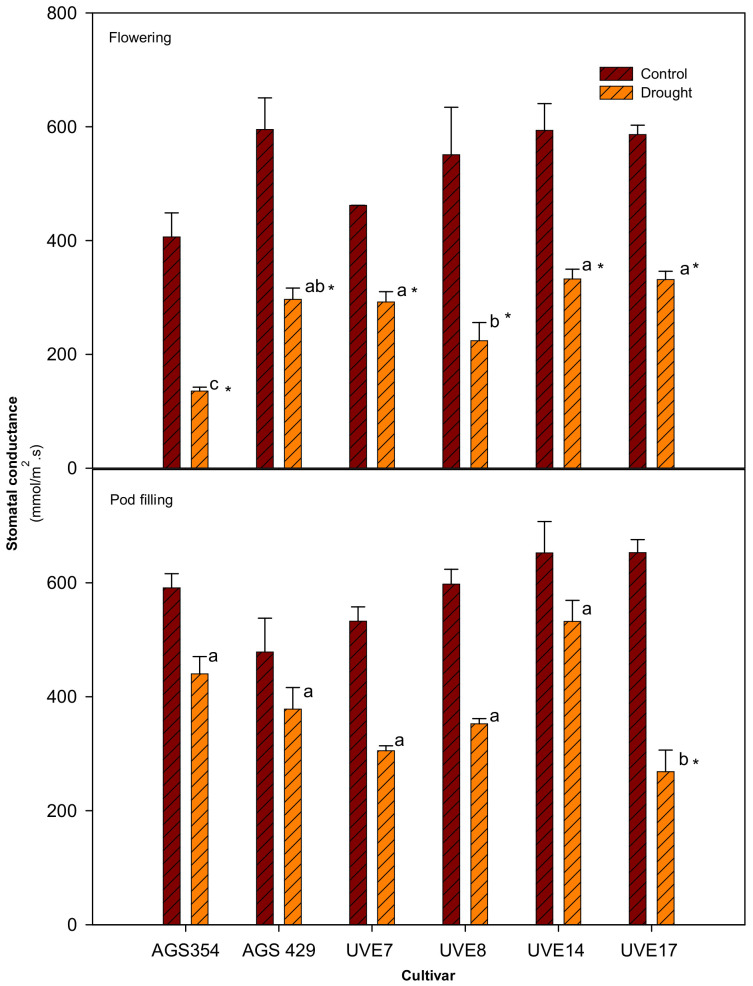
The stomatal conductance of six edamame cultivars at flowering and pod filling stages under drought stress. Letters (a, b, and c) represent differences or similarities in stomatal conductance between cultivars. Asterisk (*) represents a significant difference under drought stress relative to control. Values represent means ± SD (*n* = 3).

At flowering stage, drought stress significantly reduced the stomatal conductance of all drought-stressed cultivars. The stomatal conductance was reduced in AGS354 (by 67%), AGS429 (by 50%), UVE7 (by 36%), UVE8 (by 59%), UVE14 (by 44%) and UVE17 (by 43%) relative to control. UVE7, UVE14 and UVE17 were not significantly different from each other. AGS354, AGS429 and UVE8 were significantly different from each other and from the other cultivars. At pod filling, drought-stressed plants had higher stomatal conductance than at flowering. Cultivars were not significantly different from each other; however, UVE17 showed a decrease of 59% due to drought stress (Figure 5).

### 2.2. Non-Structured (Soluble) Sugars and Starch

**Figure 6:**
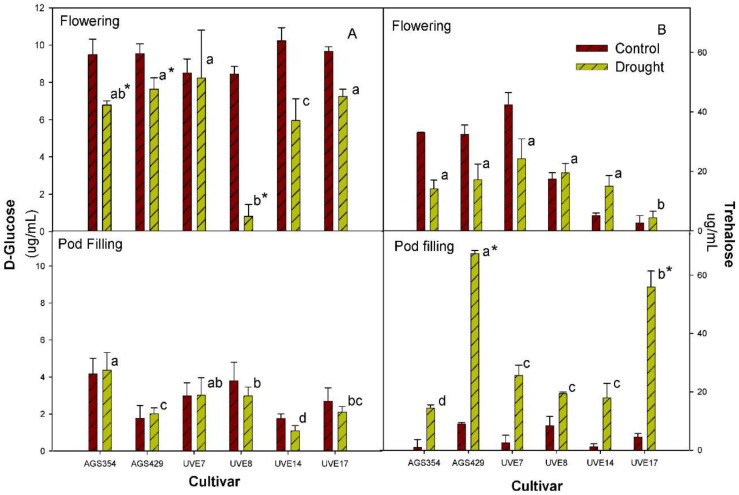

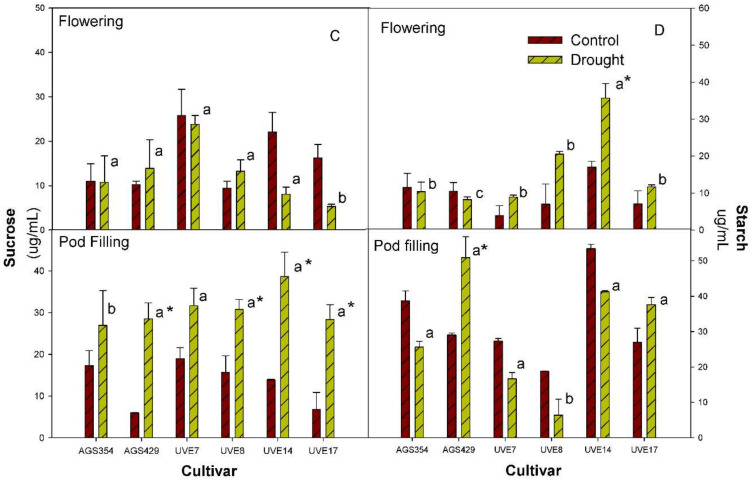
Glucose (**A**), trehalose (**B**), sucrose (**C**) and starch (**D**) content of six edamame cultivars at flowering and pod filling stages under drought stress. Letters (a, b, and c) represent differences or similarities in sugar content between cultivars. Asterisk (*) represents a significant difference under drought stress. Values represent means ± SD (*n* = 3).

Glucose was significantly reduced in all drought-stressed cultivars, except for UVE7 at flowering. The glucose content was significantly reduced by 90% in UVE8 and by 42% in UVE14 relative to control (Figure 6A). AGS354, AGS429 and UVE17 also showed significant reductions of 29%, 20% and 25%, respectively, compared to the controls. AGS429, UVE7 and UVE17 were not significantly different from each other. AGS354, UVE8 and UVE14 were significantly different from each other and from the other cultivars (Figure 6A). At pod filling, the cultivars were significantly different from each other. In addition, cultivars had lower glucose contents at pod filling compared to flowering (Figure 6A). At flowering, drought stress did not significant affect trehalose content and the cultivars were not significantly different from each other. However, UVE17 showed the lowest trehalose content. On the other hand, UVE14 showed a 3-fold higher trehalose content compared to the control (Figure 6B). At pod filling, drought stress significantly increased trehalose content in AGS429 (by 87%) and UVE17 (by 92%). Under drought stress, AGS354, AGS429 and UVE17 were significantly different from each other and from the other cultivars, whereas UVE7, UVE8 and UVE14 were not significantly different from each other. Cultivars showed a higher trehalose content at pod filling than at flowering (Figure 6B). At flowering, drought stress did not significantly affect the sucrose content for all the cultivars since the cultivars were not significantly different from each other. However, UVE17 showed the lowest sucrose content (Figure 6C). At pod filling, drought stress significantly increased the sucrose content of AGS429 (by 97%), UVE8 (by 49%), UVE14 (by 64%) and UVE17 (by 76%). AGS354 was significantly different from the other cultivars. Sucrose content was higher at pod filling than at flowering (Figure 6C). Drought stress did not significantly affect the starch content for most cultivars, but it was increased by 52% in UVE14 relative to the control. AGS354, UVE7, UVE8 and UVE17 were not significantly different from each other. AGS429 and UVE14 were significantly different from each other and from other cultivars (Figure 6D). At pod filling, drought stress reduced starch production in AGS354, UVE7, UVE8 and UVE14. However, these reductions were insignificant. In contrast, AGS429 showed a significantly higher starch content (42%) compared to the control. Only UVE8 was significantly different from the other cultivars.

**Figure 7:**
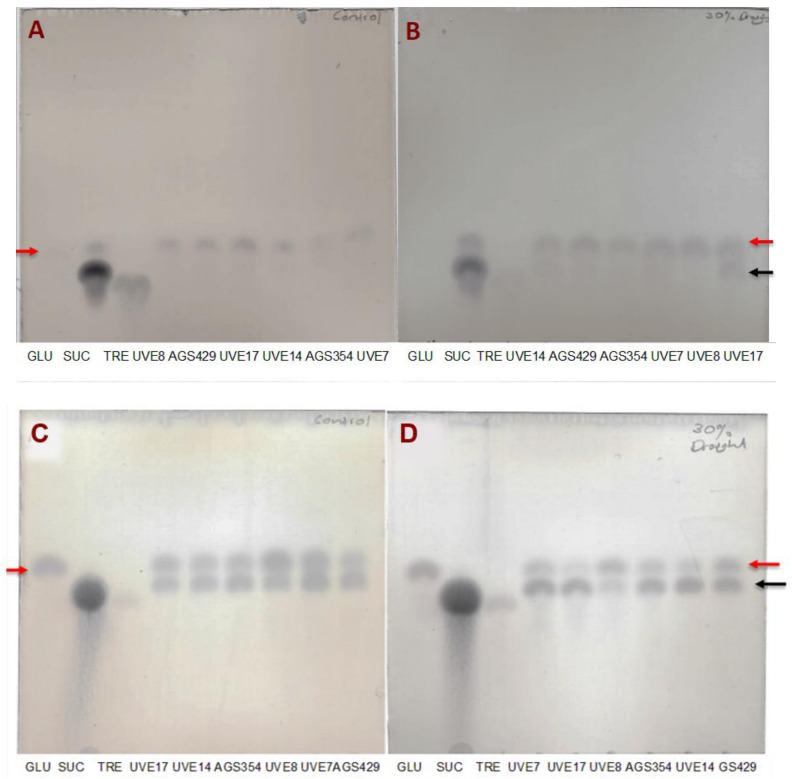
Thin-layer chromatography profiles for soluble sugars in the controls (**A**,**C**) and drought-stressed (**B**,**D**) edamame cultivars during the flowering stage (**A**,**B**) and the pod filling stage (**C**,**D**). The red arrow shows the glucose (GLU) and the black arrow represents disaccharides, such as sucrose (SUC) and trehalose (TRE).

The thin-layer chromatography (TLC) results showed that at flowering, the control cultivars only showed a single band of monosaccharide (Figure 7A), which corresponded to the glucose molecule. Except for UVE8, all drought-stressed cultivars had a second disaccharide produced, even though the bands of the disaccharide were not intense compared to the monosaccharide (Figure 7B). At pod filling, glucose intensity decreased in drought-stressed cultivars and the disaccharides spots increased in intensity. This showed an increase in sucrose/trehalose contents with a decrease in glucose content for UVE7, UVE17, AGS354 and UVE14 (Figure 7D). UVE8 contained more glucose than disaccharides. The control cultivars also showed some disaccharides contents at pod filling but the spots’ intensity was the same as for glucose (Figure 7C). Generally, the disaccharides content was higher in drought-stressed cultivars at pod filling. The control plants also produced high sucrose/trehalose at pod filling.

### 2.3. Cell Wall Parameters

**Figure 8:**
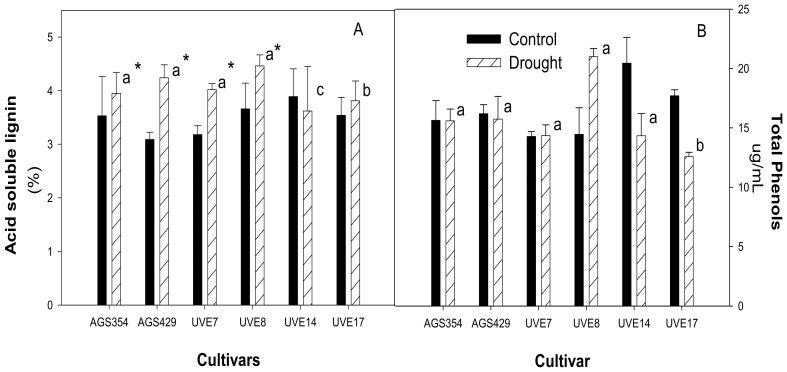
Acid soluble lignin (**A**) and total phenolic content (**B**) of six edamame cultivars at pod filling stage under drought stress. Letters (a, b, and c) represent differences or similarities in acid soluble lignin/total phenolic content between cultivars. Asterisk (*) represents a significant difference under drought stress. Values represent means ± SD (*n* = 3).

In this study, water treatment and cultivar by water treatment effects were significant (*p* ≤ 0.01 and *p* ≤ 0.05) for acid soluble lignin (ASL) (Table S1). Drought stress significantly increased soluble lignin content in the cell walls of AGS354 (by 21%), AGS429 (by 27%), UVE7 (by 20%) and UVE8 (by 17%). UVE14 and UVE17 were significantly different from each other and from the other cultivars under drought stress, whereas AGS354, AGS429, UVE7 and UVE8 were not significantly different from each other (Figure 8A). Drought stress did not significantly affect the total phenol content of all cultivars. Cultivars AGS354, AGS429, UVE7, UVE8 and UVE14 were not significantly different, except UVE17, which was significantly different from the other cultivars under drought stress (Figure 8B).

**Figure 9:**
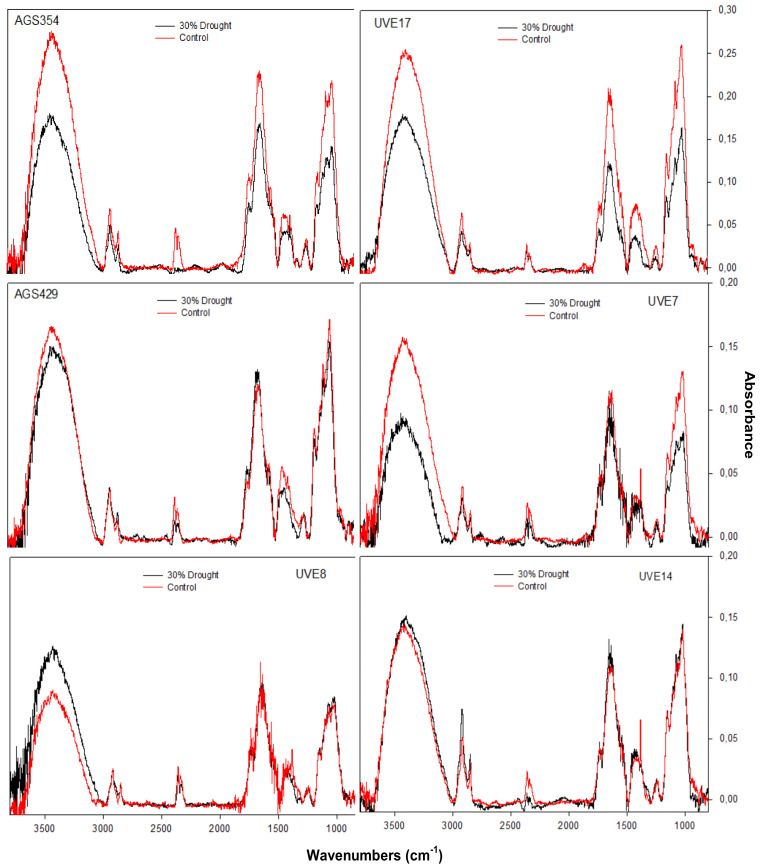
Fourier-transform infrared FTIR spectra of six edamame cultivars exposed to drought stress and the controls. Spectrum for cell wall composition was performed in the region between 4000–800 cm^−1^ at 4 cm^−1^ spectral resolution. Each sample was scanned 16× and collected in absorbance mode.

Drought stress significantly reduced the crystalline cellulose at 3800–3000 cm^−1^, lignin at 1458 cm^−1^, lignin/holocellulose at 1450–1300/1300–1000 cm^−1^, unconjugated hemicellulose at 1733 cm^−1^ and cellulose at 2850–2918 cm^−1^ of drought-stressed AGS354 and UVE17 cultivars (Figure 9). The amide (amino acid functional group) stretching at 2300 cm^−1^ was significantly reduced by drought stress in AGS354, but not significantly for UVE17. Drought stress slightly reduced the crystalline cellulose at 3800–3000 cm^−1^, amide at 2300 cm^−1^, lignin at 1458 cm^−1^ and lignin/holocellulose at 1450–1300 cm^−1^/1300–1000 cm^−1^, but the unconjugated hemicellulose at 1733 cm^−1^ and cellulose at 2850–2918 cm^−1^ regions were not significantly reduced in AGS429. In UVE7, drought stress significantly reduced the crystalline cellulose at 3800–3000 cm^−1^ and the lignin/holocellulose at 1450–1300 cm^−1^/1300–1000 cm^−1^. The amide stretching at 2300 cm^−1^ was also lower in drought-stressed UVE7. Interestingly, drought stress significantly reduced the crystalline cellulose at 3800–3000 cm^−1^, while other regions, such as the amides, lignin/holocellulose, unconjugated hemicellulose and cellulose, were not reduced in UVE8. In UVE14, drought stress slightly increased all cell wall components (lignin/holocellulose, unconjugated hemicellulose and cellulose) but reduced the amides at 2300 cm^−1^ (Figure 9).

In addition, the crystallinity index results (Table 1) demonstrated that drought stress significantly increased the crystallinity index of AGS354 by 3.3%, UVE14 by 9% and UVE17 by 12%. On the other hand, drought stress decreased the crystallinity index of AGS429, UVE7 and UVE8 by 5.6%, 4.1% and 1.3%, respectively.

Table 2 and Table 3 represent the correlations of the photosynthesis, non-structural carbohydrates cell wall contents and morphological parameters analyzed at flowering and pod filling, respectively. At flowering (Table 2), CRDs had significant positive correlations (*p* ≤ 0.05) with Chl-a, PIabs and PItotal. PI abs had a significant and strong positive correlation (*p* ≤ 0.01) with Fv/Fm and PItotal. Chl-a had a significant positive correlation with starch (*p* ≤ 0.05), while on the other hand, it had a significant negative correlation with Chl-b (*p* ≤ 0.05). The correlation between PIabs and trehalose was strong and significantly negative (*p* ≤ 0.01). At pod filling (Table 3), Chl-a showed a strong and significantly positive correlation with CRDs (*p* ≤ 0.01) and a significant positive correlation with total phenols (*p* ≤ 0.05). PIabs showed a strong and significantly positive correlation with Fv/Fm (*p* ≤ 0.01), while a significant and positive correlation between glucose and acid soluble lignin (*p* ≤ 0.05) was also observed.

## 3. Discussion

Drought is one of the most damaging abiotic stresses, and causes extensive crop loss all over the world. Linear electron transport can be monitored by using the chlorophyll fluorescence technique [34]. Drought stress selectively induced significant increases in the Fv/Fm (quantum efficiency of PSII) of AGS429 and UVE17 at pod filling (Figure 1), which could lead to an upregulation of photosynthesis according to Mathobo et al. [35]. The induction of Fv/Fm in both tolerant (AGS429) and susceptible (UVE17) cultivars indicates that it is not an important indicator of drought tolerance in edamame. The PSII quantum efficiency for the other cultivars was, however, not affected by drought stress, further showing that Fv/Fm was not an indicator of drought tolerance in edamame. Additionally, in common bean cultivars, a different plant species, Terzi et al. [36] reported that drought stress did not have any effect on the photochemistry of PSII.

The OJIP transient test of performance index on absorption basis and total performance index (PIabs and PItotal) is hypersensitive to abiotic stress and a good technique to monitor the performance of PSl and PSll [19,37]. During flowering, PIabs and PItotal were not impacted by drought stress across all cultivars (Figure 2A,B). Drought selectively induced significant increases in the PIabs of AGS429 and UVE17 at pod filling, which shows that this parameter was also not important for drought tolerance screening in edamame. In general, PIabs and PItotal were lower at pod filling compared to flowering, suggesting that the photosynthesis responses of edamame during drought stress are growth stage-specific. Jia et al. [38] also found that PSI and PSII activities declined with drought stress duration in maize. A decline in PIabs at pod filling suggests an inactivation of the PSII reaction centers (RCs) [18]. Therefore, increased PIabs in AGS429 and UVE17 under drought stress shows that these cultivars may have maintained their PSII RCs and avoided photo-inhibition of both photosystems [38]. Although cultivars generally had higher PItotal under drought stress, AGS354 and UVE17 had a decrease in PItotal at flowering and pod filling, respectively. Therefore, under drought stress, the overall performance of the photosystem was better for AGS429. A significantly positive correlation between Fv/Fm and PIabs under drought stress (Table 2 and Table 3) confirms that photosynthesis increased with an increase in quantum efficiency of PSII at both reproductive stages.

Osmotic stress resulting from drought affects the content of chlorophyll pigments [14]. The damage of chloroplasts by ROS and early leaf senescence under drought stress are the main causes of reduced chlorophyll content [36,39]. In this study, drought stress significantly reduced Chl-a content for AGS429 and UVE17 (Figure 3A), suggesting inhibited synthesis or degradation of this pigment, irrespective of the drought sensitivity of the cultivar. Chlorophyll-b is also not an indicator of drought-tolerance in edamame because, although it was reduced by drought stress in a highly susceptible cultivar (UVE17) at flowering, it increased substantially in both susceptible (AGS354) and tolerant (AGS429) cultivars at pod filling (Figure 3B). These findings agree with a report by Kalaji et al. [39], where drought stress had more devastating effects on Chl-a in *Tilia cordata*. Findings from this study were in agreement with other studies, which showed that Chl-a was more sensitive to drought stress than Chl-b [14]. Additionally, Hassanzadeh et al. [40] reported increased Chl-b, while Chl-a decreased under drought stress in sesame (*Sesamum indicum*). Furthermore, the significantly negative correlation between Chl-a and Chl-b (Table 2) showed that Chl-a decreases with an increase in Chl-b under drought stress in edamame. However, both pigments cannot be indicators of drought tolerance in edamame.

In addition to their role as accessory pigments, CRDs have antioxidative properties, which help the thylakoid and plasma membranes to become more strong and rigid under drought stress [41]. Therefore, monitoring of the CRDs content can be used as a physiological marker of plants’ drought tolerance [42]. In the current study, drought stress significantly inhibited the CRDs content of UVE17, a highly susceptible cultivar at pod filling, indicating that this cultivar could have a reduced ROS scavenging capacity, which makes it more susceptible to drought stress. In support of its role in drought tolerance, tolerant cultivars (UVE14 and AGS429) had no significant reduction in this molecule, which could translate in better growth and yield. The reduction in CRDs contents in drought-stressed ornamental cultivars was associated with reduced plant growth [42]. In edamame cultivars, low CRDs content reduced photosynthetic efficiency and resulted in poor photo-protective capacity of photosystems [43]. The significant association between CRDs and the PIabs and PItotal at flowering (Table 2) strongly suggests the protective role of CRDs on the photosystems. This confirms the importance of CRDs as indicators of drought tolerance in edamame.

In order to increase their water use efficiency during drought stress, most plants respond by closing their stomata, which reduces stomatal conductance (gs) and lowers CO_2_ intake [44]. A significant reduction in the gs at pod filling in a highly susceptible cultivar (UVE17) only (Figure 5) shows that there was less CO_2_ fixation in this cultivar. In support of this suggestion, there was less reduction in more tolerant cultivars (UVE14 and AGS429). This could be another physiological reason for its susceptibility because reduced gs means that there will be less transpiration, which could increase the leaf temperature and further inhibit photosynthesis. Similarly, early stomatal closure under drought stress was associated with a significant decrease in CO_2_ assimilation in barley [9]. The reduction of photosynthesis rate due to drought could disturb important physiological, biochemical and developmental processes, resulting in poor crop yield [45].

The non-structural carbohydrates (i.e., glucose, sucrose and trehalose) and starch serve as sources of energy for the cell, allocation of carbon in the plant cells and osmolytes [7]. The total soluble sugars content was identified to play an important role in the drought tolerance responses of edamame [3]. Drought stress significantly reduced the glucose content of all cultivars (except UVE7) at flowering (Figure 6A), which corroborated the significant reduction in gs and reduced crop yield [44]. Significant decreases in the glucose content of both susceptible (AGS354 and UVE8) and tolerant (AGS429) cultivars at the pod filling stage shows that although glucose is actively used as a vital osmoregulator [46], it is not a biochemical marker for drought tolerance in edamame. Although trehalose is effective in improving plant tolerance to drought by protecting cell membrane proteins against dehydration during water shortage [47], it cannot be a concrete biochemical marker for drought tolerance in edamame because it was substantially induced in both susceptible (UVE17) and resistant (AGS429) cultivars (Figure 6B). A significant positive correlation between PIabs and trehalose at flowering (Table 2) shows that the accumulation trehalose increases with an increase in the performance index of PSII. Sucrose accumulates in drought-stressed plants and can be hydrolyzed by invertase to produce glucose and fructose. However, drought stress may inhibit enzyme activity, which result in a high leaf sucrose content [48]. In this study, sucrose increased irrespective of the drought sensitivity of the cultivars (Figure 6C and Figure 7), thereby limiting the likelihood of this parameter from being used in edamame drought tolerance selection. Substantial increases in the starch content of drought-tolerant cultivars, UVE14 at flowering and AGS429 at pod filling, contrary to the reduction in drought-susceptible cultivars (UVE17, AGS354 and UVE8) (Figure 6D and Figure S1), strongly indicates that this molecule is important in drought tolerance responses. Starch is essential because it is degraded to replenish glucose demands in the cell during drought stress [7]. The general reduction of starch content in most edamame cultivars at flowering suggests that starch could be degraded to balance the high demands of glucose during drought due to reduced photosynthesis [49]. To further show the importance of starch in the drought tolerance response of edamame; its increase resulted in substantially increased total seed mass per plant (TSMP) at flowering (Table 2).

The plant cell wall plays a huge role in protecting cells against a wide variety of biotic and abiotic stresses [50,51]. The phenolic cross-linking between lignin and hemicellulose adds strength to cell walls [32,51]. Phenols can also act as antioxidants by donating hydrogen to detoxify ROS [52]. Previous reports suggested that the increase in lignin and total phenols is a mechanism used by drought-resistant plants to cope with stress [53]. Although drought stress significantly increased ASL in most cultivars, irrespective of their level drought sensitivity, AGS429 had the highest ASL content (27% increase), which could be better at preventing water loss [54]. According to the findings, the total phenolic (TP) content (Figure 8B) is not important in differentiating drought-tolerant from drought-susceptible cultivars because TP content was increased in almost all cultivars. These results coincide with a study by Puente-Garza et al. [53], who reported non-significant effects in the TP levels in *Agave salmiana* under drought, contrary to Naderi et al. [54], who found a significant increase in TP content in drought-stressed wheat (*Triticum aestivum*).

The cell wall biochemical and crystallinity index (CrI) analysis using FTIR and XRD provided essential information in understanding the cultivars’ response to drought stress. Drought stress significantly reduced most cell wall structural carbohydrates (cellulose and hemicellulose) in AGS354 and UVE17 (Figure 9). The crystalline cellulose, amorphous cellulose, hemicellulose, lignin and lignin/holocellulose regions of these cultivars showed significant reduction due to drought stress. The AGS354 and UVE17 holocellulolytic content measured with liquid chromatography-mass spectrometry (LC-MS) was reduced after drought stress treatment, which corroborated the FITR finding (Figure S2). However, the CrI findings from XRD (Table 1) showed that the crystallinity of microcrystalline cellulose was increased by 3.3% in AGS354 and 12% in UVE17. The crystalline cellulose is embedded in a network of amorphous polymer structure of pectin, hemicelluloses and lignin to strengthen the cell wall [26,51]. Any change that occurs to this network affects the cell wall crystallinity and analysis of crystalline cellulose [26,55,56]. This may explain increases in the CrI observations for crystalline cellulose of AGS354 and UVE17. Other cultivars that had crystalline cellulose reduced by drought stress were UVE7 and AGS429 (Figure 9, Table 1). The CrI of crystalline cellulose was significantly increased in UVE14 (Table 1), which is in line with the FTIR and LC-MS findings for this cultivar that showed increased holocellulolytic content after drought stress (Figure 9 and Figure S2). The results suggest that the plant cell wall of UVE14, followed by UVE8 and AGS429, was the most intact during drought stress.

It can be concluded that for edamame under drought stress, CRDs are important in inducing drought tolerance, since they were substantially reduced in UVE17, a drought-susceptible cultivar. Their positive relationship with the total performance index shows that they have a protective role on photosystems. In addition, the highly significant reduction in the gs of susceptible (UVE17) than tolerant (UVE14 and AGS429) cultivars, implicates the important role of this parameter in the drought tolerance responses of edamame. Furthermore, starch could be another factor that explains drought tolerance in edamame because it was induced to high levels in the tolerant (AGS429 and UVE14) cultivars but not in the susceptible cultivars (AGS354 and UVE17). Additionally, the strong positive relationship between starch and the yield parameter, TSMP, further supports this parameter as an important indicator of drought tolerance in edamame. The reduction in the hemicellulose contents of the susceptible cultivars suggest their involvement in cell wall modification that could be associated with drought tolerance in edamame. Therefore, this study highlights the importance of CRDs, gs, starch and hemicellulose as important physiological indicators of drought tolerance in edamame.

## 4. Materials and Methods

### 4.1. Plant Material and Growth Conditions

Edamame cultivars (AGS354, AGS429, UVE7, UVE8, UVE14 and UVE17) that were originally supplied by the Edamame Development Program in KwaZulu-Natal and the edamame breeding program at the University of the Free State (UFS), were germinated in seedling trays using Hygromix seedling mix (Hygrotech, RSA). Thereafter, the seedlings were transplanted in potting bags (10 L capacity) containing 10 kg sandy-loamy soil in the glasshouse (29°6′31.94″ S; 26°11′18.95″ E) at the UFS, Bloemfontein Campus, South Africa. The cultivars used were selected according to their drought sensitivities as characterized by Van der Merwe et al. [57]. AGS354 and UVE8 are high-yielding cultivars under optimal watering but highly unstable under drought stress conditions. UVE17 is a highly susceptible cultivar under drought stress. UVE14 is stable under drought stress conditions but is not a high yield performer. UVE7 is also a stable cultivar under drought stress but low yielding. AGS429 is a stable cultivar with low yield reduction under drought stress conditions.

The growth conditions were set at 25 °C during the day and 18 °C at night. Plants were watered to 100% soil water holding capacity (WHC) until drought stress was induced at three trifoliate leaf stage by withholding irrigation to reach 30% WHC. The 30% WHC was previously established to be the point of severe stress in edamame [3]. In order to maintain the water levels at the desired treatment levels, i.e., 1.6 L water for 100% WHC and 0.48 L for 30% WHC, pots were weighed daily to establish the amount of water needed. As an additional measure, Hydrosense II (Campbell scientific, Stellenbosch, RSA, sourced from US) fitted with a 12 cm sensor rod, CS659, was used to measure the volumetric water content (VWC) of the soil. For the 100% WHC, VWC was 20.9% and for the 30% WHC, VWC was at 6.12%. The trial design was a randomized, split-plot design where the main plot was the two water treatment levels and the sub-plot the cultivars. The design included three biological replications and five pots per plot.

### 4.2. Chlorophyll Fluorescence

The potential quantum efficiency of photosystem II and PSI for each cultivar was determined according to Pareek et al. [58] using a portable PEA chlorophyll fluorimeter (Hansatech Instrument, King’s Lynn, UK). The top, fully expanded young leaves were dark-adapted for 30 min, followed by illumination with excitation light energy set at 3000 μmol m^−^^2^s^−1^. The chlorophyll fluorescence was measured during two growth stages, flowering and pod filling. Photochemical efficiency of photosystem II was measured according to the following equation:

Fv/Fm = (Fm − F0)/Fm

where F0 is the minimum fluorescence, Fm is the maximum fluorescence and Fv is the variable fluorescence. The performance indexes (PIabs) were used to monitor the performance of PSI. PItotal was also used to monitor the overall photosynthetic efficiency of both photosystems. The measurements were performed between 10:00 a.m. to 12:00 p.m. during summer season at two plant growth stages (flowering and pod filling).

### 4.3. Stomatal Conductance

A leaf porometer (Li-Cor. ADC BioScientific Ltd., Hoddesdon, UK), an instrument used to quantify the humidity gradient that forms between the chamber and the surroundings of the leaf surface, was used to measure stomatal conductance (gs). The gs of the top fully expanded leaf was measured when the sun was at its peak between 10:00 a.m. to 13:00 p.m. during summer season at two plant growth stages (flowering and pod filling).

### 4.4. Pigments Extraction and Quantification

Leaf samples were collected during flowering and pod filling stages and crushed to a fine powder in liquid nitrogen and stored at −26 °C. The extraction and measurements of chlorophyll-a (Chl-a), Chl-b and carotenoids (CRDs) were performed according to Pareek et al. [58]. Briefly, 100 mg of ground tissue was homogenized in 2 mL of 80% (*v/v*) chilled acetone (Sigma-Aldrich, Saint Louis, MO, USA). The homogenate was centrifuged at 5000× *g* for 5 min at 4 °C, and the Chl-a, Chl-b, and CRDs content in the supernatant was measured spectrophotometrically using equations:

Chl-a (mg.mL^−1^) = [(12.7 × A663 nm) − (2.69 × A645 nm)]

Chl-b (mg.mL^−1^) = [(22.9 × A645 nm) − (4.68 × A663 nm)]

CRDs (mg.mL^−1^) = [(1000 × A470 nm) − ((3.27 × Chl-a) + (1.04×Chl-b))/227]

### 4.5. Determination of Non-Structural Carbohydrates in the Leaf Tissue

Soluble sugars were extracted twice from 100 mg of ground leaf tissue with 2 mL of 80% (*v/v*) ethanol (Sigma-Aldrich, RSA, Saint Louis, MO, USA) at 80 °C. Each extraction step was performed for 15 min and the mixture was centrifuged at 5000× *g* for 5 min. Activated charcoal (60 mg) (Merck, Darmstadt, Germany) was added to remove other non-sugar leaf metabolites in the supernatant by mixing and incubating the mixture at room temperature for 5 min. The mixture was centrifuged at 3000× *g* for 10 min. The contents of glucose and trehalose in the supernatant were quantified according the Megazyme (Wicklow, Ireland) GOPOD and trehalose kit, respectively. The sucrose content was quantified by modifying sucrose assays. Starch was extracted from the remaining leaf tissue pellets in the tubes according to the method described by Zhao et al. [59].

The reaction was initiated by adding 30.9 U/mg invertase (BDH biochemicals, England) to the tubes, which contain 0.05 mL of soluble sugar extraction and 0.150 mL of 50 mM sodium phosphate buffer at pH 6.5. The mixture was incubated for 15 min at 30 °C in order to catalyze the breakdown of sucrose to D-glucose and D-fructose. Glucose accumulation was quantified according to GOPOD kits (Megazyme, Co. Wicklow, Ireland). To account for the background glucose concentration and conformation of invertase activity, both the negative control (no enzyme added to the reaction) and the positive control (sucrose was used as a substrate instead of extracted samples) were included.

### 4.6. Starch Extraction and Quantification

Starch extraction was performed according to Zhao et al. [59]. The pellet in the tube (used to extract soluble sugars) was wet with 0.2 mL of 80% (*v/v*) ethanol and the tubes were stirred in a vortex mixer to aid dispersion. Then, 2 mL of 2 M KOH was added to each tube. The tubes were boiled at 100 °C for 1 h in a water bath with gentle stirring every 10 min. Thereafter, the tubes were removed and allowed to cool to room temperature before adding 8 mL of sodium acetate buffer (pH 3.8) to each sample tube for neutralizing the pH. The samples were centrifuged at 3000× *g* for 10 min and the clear supernatant was used for the determination of starch according to the Megazyme starch kit.

### 4.7. Thin-Layer Chromatography (TLC) for Soluble Sugars

Thin-layer chromatography mobile phase was composed of butanol:acetic acid:distilled water (2:1:1). Standard solutions of glucose (0.4 mg/mL), sucrose (0.5 mg/mL), trehalose (0.5 mg/mL) (Megazyme, Wicklow, Ireland) and the six samples (cultivars) were spotted on the Silica Gel 60 G F254 HPTLC plate (Merck, Darmstadt, Germany). The plate was allowed to develop for 2 h. The plate was dried and stained with 0.3% (*w/v*) Naphthol (Merck, Germany) in 95% (*v/v*) ethanol using sulfuric acid (Merck, Darmstadt, Germany) then heated with a hairdryer until all bands were visible.

### 4.8. Total Phenols and Acid Soluble Lignin

Cell wall studies were performed according to Stolle-Smits et al. [60]. Only pod filling samples were used. Since cell wall materials increased at the pod filling stage of plant development, this stage was considered realistic to represent the cell wall integrity of all cultivars.

Structural sugars (cellulose and hemicellulose) content, total phenolic (TP) content and lignin were extracted, and determined from oven-dried leaf tissues (60 mg) according to protocols by the National Renewable Energy laboratory [61]. Acid soluble lignin (%) was determined by measuring the absorbance at 320 nm and calculated using the following equation:

% Lignin = [(A320 × V × D)/ (ε × l × ODW) × 100]

where A320 represents absorbance at 320 nm, V is extract volume, D is the dilution factor, ε represents extinction coefficient at 320 nm and ODW is oven-dried weight.

Total phenolics extracted from the cell wall region were determined using Folin–Ciocalteu reagent according to Sluiter et al. [61]. A standard curve was used to determine TP content with gallic acid as the standard (Sigma-Aldrich, Shanghai, China). The absorbance of the samples and standards were measured at 700 nm.

### 4.9. Liquid Chromatography-Mass Spectrometry (LC-MS)

Two milliliters of 2 M of calcium carbonate (BDH Chemicals, England) were used to neutralize the 4% sulfuric acid extract in test tubes. The samples were filtered using 0.45 µm filtering device and then analyzed using an LC-MS. The samples were analyzed on a Shimadzu UFLC with an LC20AB binary pump, SIL20A autosampler and column oven. Separation was performed by an XBridge Amide column (250 × 4.6 mm, 3.5 µm) (Waters) using a gradient elution program between 10% acetonitrile with 0.1% ammonium hydroxide as solvent A and 90% acetonitrile with 0.1% ammonium hydroxide as solvent B over a total run time of 25 min. Following injection at 100%A the eluent composition decreased to 70%A over 15 min, followed by a drop to 60%A at 16 min, an increase to 80%A at 19 min and a further increase to 100%A up to 21 min and then left to equilibrate up to 25 min before the next sample injection. The column was kept at 40 °C for the duration of the analyses. Samples were analyzed on a Sciex 4000QTRAP mass spectrometer in negative ionization multiple reaction monitoring (MRM) mode using three transitions per analyte. For glucose the quantifying transition was 178.9 > 59.1 with qualifying transitions 178.9 > 89.1 and 178.9 > 119. The quantifying transition for xylose was 148.9 > 89.1 with qualifying transitions being 148.9 > 59.0 and 148.9 > 71.1. The source parameters were set at 15 psi curtain gas, collision gas at high, ionspray voltage at −4500 volts, heater temperature at 400 °C, nebulization gas (GS1) at 50 psi and heater gas (GS1) at 30 psi. Compound specific parameters were set as automatically optimized by the build in compound optimization wizard during continuous analyte infusion. A five-level serial dilution from 60 µg/mL to 0.006 µg/mL for each of the analytes was used as external calibrant with quantitation of the unknown sample levels from the linear range of the calibration curve. The instrument software Analyst 1.5 was used for acquisition and quantitation.

### 4.10. Fourier-Transform Infrared Spectroscopy (FTIR)

Leaf samples were dried for 72 h at 60 °C in an oven. About 1 mg of the dried tissue was mixed with 200 mg of oven-dried potassium bromide (KBr) (Sigma, France) to make a transparent disk using the finely ground mixture of a leaf sample with the aid of a hydraulic press. KBr was used as the blank during the FTIR procedure. The FTIR micro-spectroscopic imaging system used was “Nicolet Continuum Infrared Microscope” (Thermo scientific, USA), using the omnic series software. Each sample was scanned 16× and collected in absorbance mode in the 4000–800 cm^−1^ region at 4 cm^−1^ spectral resolution. Peaks’ functional groups were assigned according to Mafa et al. [26,62].

### 4.11. X-ray Diffraction (XRD) Analysis of Biomass

The Bruker diffractometer (USA) was used to do XRD using Cu Ka radiation at 40 kV and 130 mA at Coupled 2θ/Theta scanning angle and a speed of 0.5°/minute.

### 4.12. Microscopy Qualitative Analysis of Starch

Ground leaf samples were dried at 60 °C for 72 h and soluble sugars extracted from the 200 mg dried sample as described in Section 4.5. After each extraction, the tube contents were centrifuged at 5000× *g* for 5 min and supernatant discarded. An iodine solution was then prepared by dissolving 0.2 g I_2_ and 2.0 g KI (Sigma-Aldrich, Steinheim, Germany) in 100 mL distilled water. The pellets were then stained by adding 1.0 mL of iodine solution to each tube. Samples were incubated at room temperature for 30 min, centrifuged at 5000× *g* for 5 min, and pellets (which contained starch-iodine complex) were visualized under the Olympus SZX10 microscope (Olympus Life Science, Waltham, MA, USA) set at 1920 × 1200 resolution. Pictures were taken for each sample of drought-stressed and control samples.

### 4.13. Statistical Analysis

Statistical analysis for photosynthesis and cell wall parameters (except FTIR, XRD and LC-MS) was done using Genstat Release 19 software [63]. The data collected were tested for normality using the Shapiro–Wilk normality test. The logarithmic (log10) transformation was done to transform the data upon detection of skewness. The combined and separate effects of water treatments and cultivars were determined using analysis of variance (ANOVA). Means were separated using the Fischer’s protected least significant difference (LSD) test at *p* = 0.05. The relationships between photosynthesis and cell wall parameters under drought stress were done using correlations.

## Figures and Tables

**Table 1:** X-ray diffraction (XRD) analysis of cellulose crystallinity index during the pod filling stage of the six edamame cultivars under two water treatments (100% water holding capacity (WHC) and 30% WHC).

Cultivar	Crystallinity Index (%)	
	Control (100% WHC)	30% WHC
AGS354	38.7	42.0
AGS429	42.0	36.4
UVE7	38.7	34.2
UVE8	32.7	31.4
UVE14	32.1	42.0
UVE17	26.3	38.3

**Table 2:** Correlations during the flowering stage of 30% drought-stressed edamame.

	100 SM	Chl-a	Chl-b	CRDs	Fv/Fm	GLU	PIabs	PItotal	ASL	Starch	gs	SUC	TP/P	TSM/P	TS/P	TP	TRE
100 SM	-																
Chl-a	0.119	-															
Chl-b	−0.094	−0.492 *	-														
CRDs	0.146	0.503 *	0.025	-													
Fv/Fm	0.201	−0.366	0.254	−0.001	-												
GLU	0.014	−0.029	−0.002	−0.045	0.027	-											
PIabs	0.143	−0.170	0.202	0.494 *	0.684 **	0.002											
PItotal	0.167	−0.014	0.058	0.480 *	0.282	−0.000	0.810 **	-									
ASL	−0.458	0.075	−0.083	−0.131	−0.090	0.422	−0.170	−0.253	-								
Starch	0.270	0.484 *	−0.359	0.185	−0.248	−0.347	−0.087	0.064	−0.323	-							
gs	0.266	−0.176	−0.040	−0.070	−0.467	0.161	−0.234	0.137	−0.297	0.183	-						
SUC	−0.265	−0.189	0.208	−0.253	−0.262	−0.097	−0.394	−0.105	0.0913	−0.085	0.225	-					
TPP	−0.137	−0.131	−0.174	−0.354	−0.042	−0.193	−0.165	−0.145	0.318	−0.313	−0.317	0.027					
TSMP	−0.114	−0.490 *	0.199	−0.427	0.163	−0.060	−0.109	−0.240	−0.008	0.62 **	0.026	0.201	0.266	-			
TSP	0.129	−0.095	0.183	−0.308	0.050	−0.227	−0.187	−0.232	−0.059	−0.014	0.121	0.186	0.036	0.553 *	-		
TP	−0.448	0.114	0.152	0.162	−0.021	0.010	0.147	0.136	−0.137	−0.103	−0.289	−0.118	−0.075	0.0311	−0.22	-	
TRE	−0.390	0.208	−0.280	−0.434	−0.332	0.186	−0.628 **	−0.391	0.121	0.054	0.079	0.503	−0.112	0.060	0.017	0.19	-

Number of observations = 15. * *p* ≤ 0.05, ** *p* ≤ 0.01, 100 SM = 100 Seed mass, Chl-a = Chlorophyll-a, Chl-b = Chlorophyll-b, CRDs = Carotenoids, Fv/Fm = Ratio of variable fluorescence to maximal fluorescence of PSll, PIabs = Performance index of PSl and PSll, PItotal = Total performance index of PSl and PSll, ASL = Acid soluble lignin, gs = Stomatal conductance, TP/P = Total pods per plant, TSM/P = Total seed mass per plant, TS/P = Total seeds per plant, TP = Total phenols, TRE = Trehalose, GLU = Glucose, SUC = Sucrose.

**Table 3:** Correlations during pod filling stage of 30% drought-stressed edamame. Number of observations = 15.

	100 SM	Chl-a	Chl-b	CRDs	Fv/Fm	GLU	PIabs	PItotal	ASL	Starch	gs	SUC	TP/P	TSM/P	TS/P	TP	TRE
100 SM	-																
Chl-a	−0.513 *	-															
Chl-b	0.259	−0.389	-														
CRDs	−0.424	0.87 **	0.010	-													
Fv/Fm	−0.040	−0.236	0.125	−0.2014	-												
GLU	−0.308	0.225	−0.079	0.204	−0.162	-											
PIabs	0.234	−0.465	0.108	−0.386	0.874 **	−0.401	-										
PItotal	0.244	−0.012	−0.156	−0.163	0.098	−0.383	0.210	-									
ASL	−0.458	−0.021	0.105	0.0566	−0.252	0.487 *	−0.366	−0.232	-								
Starch	0.360	−0.415	0.442	−0.311	0.159	−0.139	0.231	0.206	−0.176	-							
gs	0.370	0.118	−0.100	0.068	0.237	0.278	0.124	0.038	−0.298	0.174	-						
SUC	0.064	−0.027	−0.057	−0.012	0.035	−0.410	0.171	0.407	−0.281	0.082	−0.325	-					
TP/P	−0.137	−0.315	0.035	−0.431	0.196	0.413	0.079	−0.186	0.318	0.060	0.045	−0.098	-				
TSM/P	−0.114	−0.080	−0.234	−0.219	0.056	0.155	0.049	−0.047	−0.008	0.219	0.085	−0.407	0.266	-			
TS/P	0.129	−0.220	−0.295	−0.304	0.146	−0.029	0.212	0.253	−0.059	0.322	0.270	0.110	0.036	0.553 *	-		
TP	−0.447	0.588 *	−0.149	0.459	0.199	−0.003	0.026	−0.126	−0.137	−0.133	−0.091	−0.035	−0.075	0.031	−0.22	-	
TRE	0.057	−0.449	0.173	−0.391	0.146	−0.252	0.243	0.080	0.167	0.402	−0.116	−0.367	−0.166	0.450	0.286	−0.327	-

Number of observations = 15. * *p* ≤ 0.05, ** *p* ≤ 0.01, 100 SM = 100 Seed mass, Chl-a = Chlorophyll-a, Chl-b = Chlorophyll-b, CRDs = Carotenoids, Fv/Fm = Ratio of variable fluorescence to maximal fluorescence of PSll, PIabs = Performance index of PSl and PSll, PItotal = Total performance index of PSl and PSll, ASL = Acid soluble lignin, gs = Stomatal conductance, TP/P = Total pods per plant, TSM/P = Total seed mass per plant, TS/P = Total seeds per plant, TP = Total phenols, TRE = Trehalose, GLU = Glucose, SUC = Sucrose.

## Data Availability

Data is contained within the article and Supplementary Materials.

## Supplementary Materials

The following supporting information can be downloaded at: https://www.mdpi.com/article/10.3390/plants11030394/s1, Figure S1: The iodine-starch complexes visualized with light microscopic for qualitative determination of starch content in control and 30% drought-stressed edamame cultivars. The intense dark blue color represents higher starch content concentration and the mixture of blue and pale color represents a lower starch content concentration. AGS354, UVE8, AGS429, UVE14, UVE7 and UVE17 control and drought-stressed cultivars are shown in the figure; Figure S2: The cell wall sugars percentage (Xylose represents the side chains of hemicellulose (A) and Glucose represents cellulose content (B)) of six edamame cultivars under drought stress. Values represent means ± SD (*n* = 2 technical replications); Table S1: Analysis of variance for the cell wall parameters during the pod filling stage of the six edamame cultivars under two water treatments (100% water holding capacity (WHC) and 30% WHC).
