# Supplementary material for: The Photosynthetic Efficiency and Carbohydrates Responses of Six Edamame (Glycine max. L. Merrill) Cultivars under Drought Stress
Source: Plants (Basel). 2022 Jan 31;11(3):394. doi: 10.3390/plants11030394 (PMC8840725; doi:10.3390/plants11030394)
Supplement: Supplementary file 1 [file plants-11-00394-s001.zip › plants-1533767-supplementary.pdf]

plants

Supplementary Materials:

Table S1. Analysis of variance for the cell wall parameters during the pod filling stage of the six edamame cultivars under two water treatments (100% water holding capacity (WHC) and 30% WHC).

	Cultivar (C)	Treatment (T)	CxT
**ASL**	0.2414	2.4620**	0.7018*
**TP**	0.415	0.265	1.224
**CV%**	13.22	80.77	27.11
**Grand Mean**	0.6564	1.36	0.9629

*$p \leq 0.05$, **$p \leq 0.01$, ***$p \leq 0.001$, ASL = Acid soluble lignin, TP = Total phenols, CV = Coefficient of variation

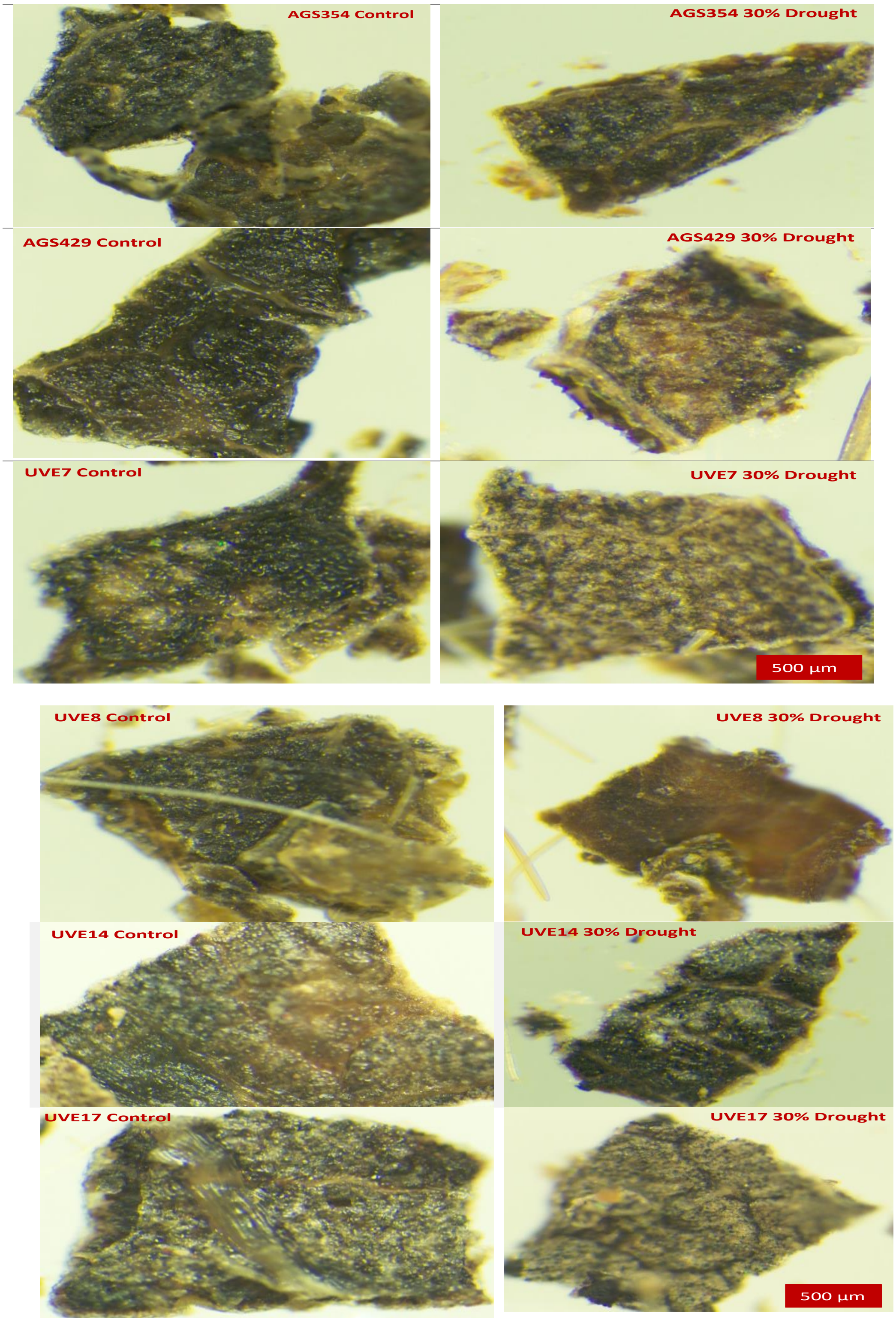

Figure S1. The iodine-starch complexes visualized with light microscopic for qualitative determination of starch content in control and 30% drought-stressed edamame cultivars. The intense dark blue color represents higher starch content concentration and the mixture of blue and pale color represents a lower starch content concentration. AGS354, UVE8, AGS429, UVE14, UVE7 and UVE17 control and drought-stressed cultivars are shown in the figure.

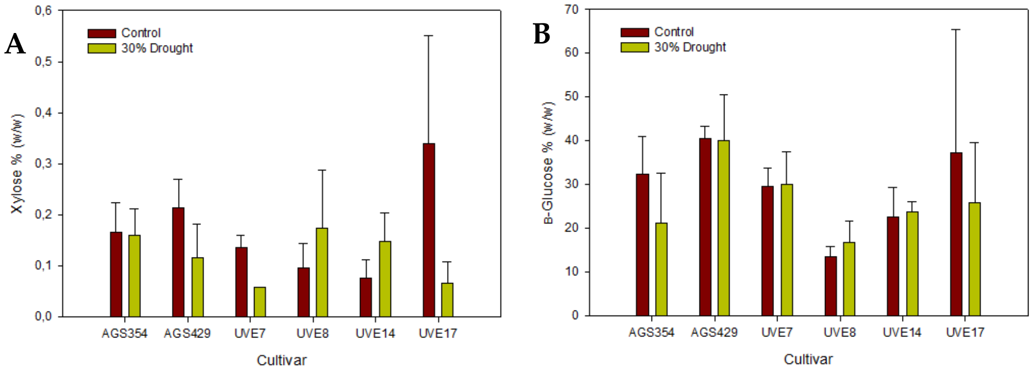

Figure S2. The cell wall sugars percentage (Xylose represents the side chains of hemicellulose (**A**) and Glucose represents cellulose content (**B**)) of six edamame cultivars under drought stress. Values represent means ± SD (n = 2 technical replications).
